# Relationships between psychopathological symptoms, pandemic-related stress, perceived social support, and COVID-19 infection history: a network analysis in Chinese college students

**DOI:** 10.3389/fpsyt.2024.1340101

**Published:** 2024-02-02

**Authors:** Chi Kei Krystal Lee, Kwun Nam Joe Chan, Sau Man Corine Wong, Hou Sem Gabbie Wong, Hiu Ching Janet Lei, Yuen Kiu So, Shi Cheng Vivian Fung, Sai Ting Ryan Chu, Kar Kin Albert Chung, Pak Wing Calvin Cheng, Ka Ying Heidi Lo, Wai Chi Chan, Wing Chung Chang

**Affiliations:** ^1^ Department of Psychiatry, School of Clinical Medicine, Li Ka Shing (LKS) Faculty of Medicine, The University of Hong Kong, Hong Kong, Hong Kong SAR, China; ^2^ School of Public Health, Li Ka Shing Faculty of Medicine, The University of Hong Kong, Hong Kong, Hong Kong SAR, China; ^3^ State Key Laboratory of Brain and Cognitive Sciences, The University of Hong Kong, Hong Kong, Hong Kong SAR, China

**Keywords:** network analysis, social support, depression, anxiety, COVID-19

## Abstract

**Introduction:**

Previous coronavirus, 2019 (COVID-19) research has applied network analysis to examine relationships between psychopathological symptoms but rarely extended to potential risk and protective factors or the influence of COVID-19 infection history. This study examined complex inter-relationships between psychopathological symptoms, COVID-19–related stressors, perceived social support, and COVID-19 infection history among Chinese university/college students during the peak of fifth pandemic wave using a network analysis approach.

**Methods:**

A Least Absolute Shrinkage and Selection Operator–regularized partial correlation network using Gaussian graphical model was constructed in 1,395 Chinese university/college students in Hong Kong who completed a survey between 15 March and 3 April, 2022. Depressive, anxiety, and acute/traumatic stress symptoms were measured by Patient Health Questionnaire-9, Generalized Anxiety Disorder-7, and Impact of Event Scale-6, respectively. COVID-19–related stressors and perceived social support were measured. Network differences by COVID-19 infection history (COVID-network vs. no_COVID-network) and network communities were examined.

**Results:**

Our results showed that the most influential nodes were depressed mood, uncontrollable worries, and uncontrollable thoughts about COVID-19. The main bridging symptoms were concentration problems and psychomotor problems. The COVID-network, comprising participants with a history of COVID-19 infection only, was significantly stronger than the no_COVID-network. Perceived social support and stress from conflicts with family/friends formed a unique community with negative cognition and suicidal idea in the COVID-network only.

**Conclusion:**

Our findings indicate that specific interventions targeting interpersonal conflicts and concentration problems as well as facilitating stress buffering effects of social support may represent effective strategies to reduce psychological distress in university/college students during COVID-19 and should be considered for future pandemic preparedness.

## Introduction

Studies on network analysis of psychopathological symptoms gained popularity before the coronavirus, 2019 (COVID-19) pandemic (1). This analytic approach was grounded on the hypothesis that psychiatric symptoms were mutually reinforcing and they cohered to form mental disorders (2). Instead of reducing mental disorders into its presence or absence as in most epidemiological studies, researchers studied the relationships among the symptoms and the process of how activation of one may activate other symptoms, leading to development of a disorder or comorbidity. The hypothesis assumes that interventions targeting the more influential symptoms may be effective in preventing the occurrence of a cascade of symptoms in highly connected networks (3). In the context where psychiatric symptoms are prevalent in the community during a global crisis like the COVID-19 pandemic, interventions targeting a few important symptoms may be easier to deliver and more effective for public mental health promotion.

Several network analyses on common psychopathological symptoms during COVID-19 have been published (4–6). Results were heterogenous because each study used different scales for a single or combination of disorders at different stages of the pandemic. A few extended the analyses to explore the relationship of the symptom network to other variables like academic performance in university students (7) or economic stress in adult population (8). Some investigated whether COVID-19 infection affected the symptom network. Zavlis et al. (2022) showed that the infection was transiently related to flashbacks in a sample with low COVID-19 prevalence (8), and no significant difference was reported by Ventura-León et al. (2022) whose networks only included two depressive symptoms (7).

Evidence to date confirmed the increase in common mental disorders during the COVID-19 pandemic and its decline subsequently. The rise was directly associated with the average number of daily COVID-19 cases and periods of the social restriction regulations (9, 10). University students constituted one of the most affected groups who faced multiple disruptions to both their academic and social life. Most studies showed that at least half of them had significant depressive, anxiety, or acute/traumatic stress symptoms (10, 11). From studies of university students during the COVID-19 pandemic, the reported prevalence of anxiety ranged from 29% to 32%, depression ranged from 34% to 43%, and acute stress up to 67% (12, 13). Numerous risk factors like having physical or psychiatric conditions, being infected with COVID-19, having high levels of COVID-19–related concerns and protective factors such as frequent exercise have been reported (14, 15). Among the protective factors, social support, commonly understood as the information, assistance, or comfort arising from social relationships to help individuals to deal with stressors, was the most important one as identified in a meta-analysis (15, 16).

The present study was conceptualized primarily to understand the most common psychopathological symptoms, i.e., depressive, anxiety, and acute/traumatic stress symptoms and their relationships to perceived social support and psychosocial stressors using a network approach in a group of university/college students sampled during the peak of the COVID-19 pandemic in early, 2022 in Hong Kong (HK). It was a time when the number of infections soared to an unprecedented level with the highest worldwide death record after a prolonged period of zero-transmission policy with highly stringent social distancing and testing regulations (17, 18). We planned to characterize the pathways among the COVID-19–related stressors, the social support, and the psychopathology symptoms to derive insights for the prevention of negative mental health symptoms in a pandemic situation. Our second objective was to explore any network differences between those with and without COVID-19 infection. This was considered important because individuals with COVID-19 infection could have experienced physical symptoms like lethargy and poor appetite that mimicked depressive symptoms (19, 20); they could have faced unique stress from physical symptoms or prolonged isolation and exhibited different patterns of psychiatric symptoms. The null hypothesis was that the psychopathology networks in those with and without COVID-19 infection were not significantly different in terms of strength and edge differences.

## Materials and methods

### Study sample and procedures

The sample was derived from a cross-sectional survey conducted for non-institutionalized adults aged 18 years or above in HK from 15 March to 3 April, 2022. The methodology has been described elsewhere (21). A virtual snowballing sampling technique, a method commonly used during COVID-19 when social distancing measures were in place (22–24), was adopted for data collection, with an online anonymous self-rated questionnaire being administered on a Qualtrics survey platform (https://www.qualtrics.com). This platform was chosen because of its comprehensive security protection, versatility in survey settings, question formats, and response options (25). The survey was disseminated through social media platforms (e.g., Facebook, Instagram, Twitter, and WhatsApp), universities (via emails), and the HK Public Opinion Research Institute (HKPORI), a well-established survey agency executing independent public surveys for academic institutions and government departments by sending email invitations with survey link to the members of its probability- and non-probability–based online panels of adult local residents. Respondents were encouraged to forward the survey link to their social networks for study participation. Survey participation was voluntary, and an informed consent was obtained before the questionnaire assessment. In the current study, a response was included for analysis if the respondents reported being aged 18–30 years, were full-time university/college students, able to read and understand Chinese, and resided in HK at the time of survey. Those who did not provide consent or failed to complete the questionnaire items on the core measures of the current study (i.e., all psychopathology scales, COVID-19–related stressors, COVID-19 infection status, and perceived social support) were excluded. The study was approved by the Institutional Review Board of the University of Hong Kong/Hospital Authority Hong Kong West Cluster (HKU/HA HKW). A total of 1,359 respondents fulfilled the inclusion criteria and constituted the final sample for analysis.

### Study assessments

#### Psychopathological symptom measures

Three major psychopathological symptoms including depressive, anxiety, and acute/traumatic stress symptoms were evaluated because they were the most prevalent symptoms reported in university students during COVID-19 pandemic (10–13). The Patient Health Questionnaire-9 (PHQ-9) (26, 27) assessed depressive symptom severity during the past 2 weeks according to the nine symptoms in the DSM-IV on a four-point Likert scale (from “not at all” to “nearly every day”). Good internal consistency and convergent validity have been reported in young people in HK (28). The General Anxiety Disorder-7 (GAD-7) (27, 29) assesses anxiety symptom severity during the past 2 weeks with seven items rated on a four-point Likert scale (from “not at all” to “nearly every day”). The GAD-7 has shown good internal consistency and convergent validity in a pre–COVID-19 youth sample (30). The Chinese version of GAD-7 has been validated (31). Acute/traumatic stress symptoms on a four-point Likert scale were measured by Impact of Event Scale-6 (IES-6), a six-item tool evaluating stress symptoms occurred in the last week (32). Higher scores indicate more severe symptoms. The Chinese version has been used in various studies (33, 34). The Cronbach’s alpha values for PHQ-9, GAD-7, and IES-6 in this sample were 0.89, 0.93, and 0.86, respectively.

#### COVID-19–related stressors, perceived social support, and COVID-19 infection status

The experience of COVID-19–related stressors was assessed by ratings on a five-point Likert scale [0 (not stressful) to 4 (extremely stressful)] on the following domains: finance, work, physical health, food and supplies, medical care and medication, family relationship (e.g., verbal or physical conflicts in family), and interpersonal relationships (e.g., verbal or physical conflicts among friends). A single-item subjective stress level was found to be valid and correlated with various mental health outcomes in a pre–COVID-19 youth sample in HK (35). The stressors were recategorized to reduce the numbers of highly correlated items in the network, i.e., access to medical care, food or supplies (r = 0.67), finance or work stress (r = 0.57), and family or interpersonal conflicts (r = 0.47) by taking the mean of the scores from both domains. The stressor ratings were collapsed into three levels (0, not stressful; 1, a little stressful; 2 or more, stressful to extremely stressful) to optimize the number of observations in each level. Perceived social support from family and friends (SSFm and SSFr, respectively) were measured on a seven-point Likert scale, respectively. A single-item question on social support was demonstrated to be valid among university students pre–COVID-19 (36) and was used in other studies during the COVID-19 pandemic (37). Lastly, subjects were asked to report whether they had any history of COVID-19 infection. Self-reporting of COVID-19 infection history was used in other similar studies (7, 8).

### Statistical analysis

#### Network estimation

All variables were incorporated in a partial correlation network using LASSO regularization (Least Absolute Shrinkage and Selection Operator) (38, 39). A network was selected using Extended Bayesian Information Criterion (EBIC) with γ = 0.5 to minimize the false-positive associations to zero, resulting in a sparse network allowing easier interpretation (39, 40). The model was estimated using the bootnet R-package (version 1.5) using “EBICglasso” method (39, 41). Spearman correlations were used to obtain more stable estimates (39, 42).

The network was visualized using qgraph R-package (version 1.9.4) as a set of nodes, representing the variables, and their interconnecting lines, representing their relationships (39, 43). The thickness of the lines or edges captures the partial correlations that is the correlation between the two variables when controlling for all other items shown. The edge weights, ranging from −1 to 1, indicate the direction and strength of the partial correlations between two nodes. Network colors were fixed with blue and red indicating positive and negative edge weights, respectively. Negative edges were dashed.

#### Network inference and predictability

Recent literature has suggested that several centrality measures including closeness and betweenness are unreliable and unsuitable in assessing nodes’ importance in psychopathology networks, and strength is inappropriate if there are both positive and negative edges in the networks (44–46). In this study, expected influence was computed using qgraph R-package to evaluate the importance of individual node in the network taking into account the valence and weight of its edges (46). Node predictability was computed to quantify the variance of each node explained by its neighboring nodes using mgm R-package (version 1.2-13) (47). It is plotted as a pie chart in the outer ring of each node using qgraph R-package.

#### Network accuracy and stability

Bootstrapping was applied to centrality index, i.e., expected influence and edge-weight parameters using bootnet R-package to examine network stability and accuracy. It was applied to evaluate the stability of centrality indices by case-dropping. The indices were repeatedly calculated with different subsets of data that consisted of different proportions of data dropped. Stability was evaluated by the correlation stability coefficient (CS-coefficient), which is the maximum proportion of cases that could be dropped with a 95% certainty. A CS-coefficient above 0.5 indicates a good stability (41). Edge-weight accuracy was assessed by calculating their confidence intervals derived from 1,000 non-parametric bootstrap samples. Bootstrapped differences tests were conducted to test for significant differences in edge weights and node centrality using a set of 1,000 bootstrapped samples.

#### Network comparison and network communities

A comparison of the networks between those with and without a report of COVID-19 infection history (COVID network and no_COVID network) was performed using the NetworkComparisonTest (NCT) R-package with a permutation seed value of “123” (48). On the basis of 1,000 permutations, the invariant network structure, invariant edge strength, and invariant global strength were investigated. The Bonferroni–Holm procedure, a powerful method to control the family-wise error rate commonly used in comparing network structures, was used for multiple comparisons in the current study (5, 48).

Exploratory graph analysis using EGAnet R-package (version 1.2.3) with walktrap algorithm was carried out to identify the optimal number of subnetworks within each network (49, 50). The walktrap algorithm conducts random walks over the estimated network, forms boundaries between nodes, and forms communities of clusters of highly connected nodes deterministically (49, 51). These subnetworks represent patterns of strong interconnectivity among the nodes within the network. Fitness of the model was evaluated using confirmatory factor analysis in the EGAnet R-package (50, 52). “Bridge expected influence” was estimated using networktools R-package (version 1.5.0) to identify the most influential nodes, bridging across these subnetworks by summation of the value of all edges connecting a specific node with nodes in the other community (50, 53).

## Results

### Characteristics of the sample

Sample characteristics are shown in **Table 1**. There were more women (64.9%) in the sample with 30.7% reporting a history of COVID-19 infection. Fifty-six percent met the criteria for either probable depression, anxiety, or post-traumatic stress disorder (PTSD) defined as scoring 10 or more in the respective scales. Those reporting a history of COVID-19 infection had increased odds for being probable cases of depression [odds ratio (OR), 1.56; p < 0.01), anxiety (OR, 1.53; p < 0.01), and PTSD (OR, 1.26; p = 0.04), but they did not differ significantly in individual symptom score. The distribution of the items in all scales is shown in **Supplementary Tables S1**, **S2**.

**Table 1:** Demographics, perceived social support, mental health distress, and COVID-19–related stress levels in the study sample (N = 1,359).

	Number (%)^a^
Demographic and illness profile	
Male sex	477 (35.1)
Substance or alcohol abuse	18 (1.3)
History of psychiatric illness	99 (7.3)
History of COVID-19 infection	319 (30.7)
Perceived social support	
Social support from family, mean (SD)	3.36 (1.75)
Social support from friends, mean (SD)	4.25 (1.35)
Mental health distress^b^	
Probable depression	540 (39.7)
Probable anxiety	400 (29.4)
Probable PTSD	515 (37.9)
Either probable depression, anxiety, or PTSD	757 (55.7)
COVID-19–related perceived stress^c^	
Finance or work	630 (46.3)
Physical condition	694 (51.6)
Getting medical attention, food, or other materials	489 (36.0)
Conflicts with family or friends	458 (33.7)

COVID, coronavirus; PTSD, post-traumatic stress disorder; SD, standard deviation.

^a^ Data are presented in number and percentage, unless otherwise specified.

^b^ Probable depression, probable anxiety, and probable PTSD are defined as PHQ-9 score, GAD-7 score, IES-6 score ≥10, respectively.

^c^ Numbers reported stressful to extremely stressful [2 or above in a five-point (0–4) Likert scale] in the domain. The mean score was used if the stressor involved more than one correlated domain.

### Network structure and analyses

**Figure 1:**
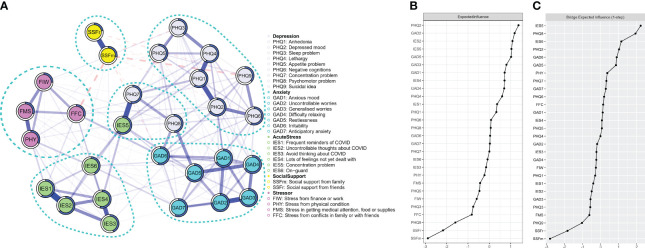
**(A)** Regularized partial correlation network model of depressive, anxiety, and stress symptoms; COVID-19–related stressors; and perceived social support in a sample of university/college students in Hong Kong during the peak of COVID-19 pandemic. Blue lines between two nodes indicate positive correlations, and dotted red lines indicate negative correlations. Predictability of each node is represented by the circles surrounding each node. **(B)** Expected influence of each node in the network. **(C)** Bridge expected influence in the network. X-axis represents the z-scores.

**Figure 1A** showed the network of three sets of psychopathological symptoms, COVID-19 stressors, and perceived social support in the sample. The corresponding partial correlation matrix is shown in **Supplementary Table S3**. All nodes within individual domains are quite well connected with several positive connections between the depression and anxiety symptoms domains. The strongest connection between the stressors and symptoms was stress from conflicts with family or friends (FFCs) and irritability (GAD-6) (r = 0.10). SSFm was negatively connected with suicidal ideation (PHQ-9) (r = −0.09), sleep problem (PHQ-3) (r = −0.05), and irritability (GAD-6) (r = −0.05). SSFr was negatively connected with suicidal idea (PHQ-9) (r = −0.05)

### Network inference and predictability

Depressed mood (PHQ-2), uncontrollable worries (GAD-2), and uncontrollable thoughts about COVID-19 (IES-2) showed the highest node expected influence (**Figure 1B**). The mean predictability across all nodes was 0.50, indicating that, on average, half of the variance of a node can be explained by its neighboring nodes in the network. The average predictability of depressive, anxiety, and acute stress symptoms were 0.51, 0.65, and 0.54, respectively (**Supplementary Table S1**).

### Network stability and accuracy

Bootstrapped 95% CIs showed a narrow curve, suggesting reliable and accurate edge-weight estimates (**Supplementary Figure S1**). Results from the bootstrapped difference tests (**Supplementary Figures S2**, **S3**) revealed that most edge weights and node strengths were statistically different from one another in the resulted network. CS-coefficient was 0.75 for expected influence, indicating a good network stability in this regard (**Supplementary Figure S4**).

### Network comparisons

**Figure 2:**
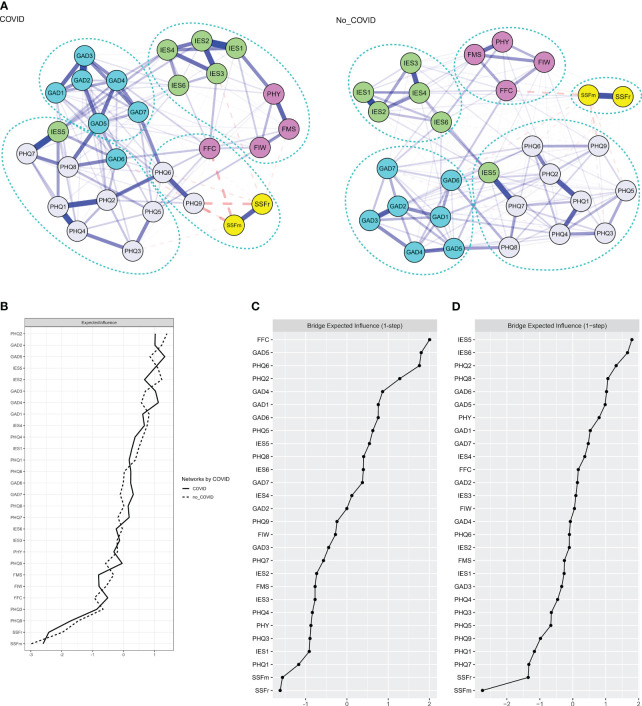
**(A)** Regularized partial correlation network model of depressive, anxiety, and acute stress symptoms; COVID-19 stressors; and perceived social support in a sample of university/college students in Hong Kong during the peak of COVID-19 pandemic by their reported history of COVID-19 infection. Blue lines between two nodes indicate positive correlations and dotted red lines indicate negative correlations. **(B)** Expected influence of each node in the network. **(C, D)** One-step bridge expected influence in the COVID and no_COVID networks. X-axis represents the z-scores.

Network comparison tests revealed a significant difference in global strength between the COVID and no_COVID networks with respective global strength of 13.07 and 12.06 (S = 1.00, p = 0.01) (**Figure 2A**). Edges with significant strength difference are shown in **Table 2**. There was no significant difference in network invariance (M = 0.16, p = 0.30) or expected influence of individual nodes (**Figure 2B**). The corresponding partial correlation matrices are shown in **Supplementary Table S4**, **S5**.

**Table 2:** Significant edge differences between networks consisting of participants with and without report of COVID-19 infection^a^.

Edges with between-network differences		Partial correlation coefficient of two nodes in each network	
**Node 1**	Node 2	COVID network	No_COVID network
**PHQ-9**	GAD-1	0.073	0.00
**GAD-4**	GAD-7	0.160	0.00
**GAD-4**	IES-4	0.085	0.00
**IES-3**	SSFr	0.045	0.00
**IES-1**	PHY	0.169	0.04
**PHQ-5**	FFC	0.064	0.00

COVID, coronavirus; GAD, Generalized Anxiety Disorder-7 Scale; FFC, conflicts with family or friends; IES, Impact of Event Scale; PHQ, Patient Health Questionnaire; PHY, stress from physical condition; SSFr, perceived social support from friends.

^a^ Adjustments for multiple comparisons were made using Holm–Bonferroni method.

### Network communities and bridging nodes

Communities identified in the exploratory graph analysis of the overall, COVID, and no_COVID networks are shown in **Figures 1A**, **2A**. Confirmatory factor analysis confirmed that all models had satisfactory to good fitness indexes (**Supplementary Table S6**). Only in the COVID network, SSFm and SSFr formed a distinct community with negative cognition (PHQ-6), suicidal idea (PHQ-9), and stress from FFCs. The other stressors coalesced with most IES-6 symptoms to form a separate community. All stressors and perceived social support were separated from the symptom communities in the overall and no_COVID network. The bridge expected influences of all networks are shown in **Figures 1C**, **2C, D**.

## Discussion

To the best of our knowledge, this is the first study exploring the relationships among perceived social support, COVID-19 stressors, and symptoms of three common mental disorders using a network analysis approach. Compared with the previous studies that incorporated an isolated stressor (8) general measure of social support without referring to the source (54) or single global measure of mental health or quality of life (37) during COVID-19, our study encompassed comprehensive appreciation of potential sources of support and stressors and their specific relationships to a wide range of common psychiatric symptoms during the peak of COVID-19 pandemic. Our results were stable and robust and the overall symptom network structures were comparable to previous studies. Notably, our findings revealed an important external connection of stress from FFCs in activating the whole network of psychopathological symptoms, particularly in those infected with COVID-19. The relationships between the perceived social support and specific symptoms and its influence over the overall network were demonstrated. These observations were discussed in detail below.

Perceived SSFm and SSFr, to a lesser extent, were crucial in deactivating the network of a range of interrelated common psychiatric symptoms as reflected by the negative expected influence of these nodes. Specifically, suicidal ideation, irritability, and sleep problems had stronger negative correlations with social support especially from family. This observation was consistent with extensive literature on the importance of support from family over friends, particularly in younger populations, and its negative association with suicidal ideation, depressive, and anxiety symptoms (55–57). Unexpectedly, we did not find any negative but small positive connections between perceived SSFr and SSFm and acute stress symptoms, in contrast with the stress buffering effect of social support (58, 59). At the time of survey, social distancing measures were at the strictest level. All schools and recreational facilities had been closed for 3 months, and gatherings of more than two individuals were prohibited in public places. It was possible that individuals did not access the perceived support they had in reality, as hypothesized by Szkody et al. (2021) (60). In addition, local and social media were fed with news about the breakdown of public health system and panic-buying (61, 62). Perceived social support may have included the spread of fear or biased information, leading to the negative effects on mental health as similarly reported by Li et al. (2023) (63). During an unparalleled crisis, those with higher stress levels might have reached out for more support that was yet to fully meet their needs or improve their sense of control (64).

Our study revealed the pattern of connections between common stressors and psychopathological symptoms. Connections between the stressors were more consistent with most acute/traumatic stress symptoms and more scattered with anxiety or depressive symptoms. The observation was reasonable as the stressors were likely acute in nature. Anxiety and depressive symptoms were more related to chronic and cumulative stress experienced in vulnerable groups. A point to note is the strong connection between stress from FFCs and irritability, similarly reported elsewhere during the pandemic (65). This relationship could be bidirectional, meaning the irritability is a consequence or cause for interpersonal conflicts. The impact from stress from finance or work might be less immediate in our sample comprising of full-time students.

Our study demonstrated significant differences among those with and without history of COVID-19 infection. The stronger connectivity in the COVID network could be related to the more severe symptoms in the group (1). Stress from physical condition was more connected with “other things” keep reminding me of COVID-19 (IES-1) in the COVID network. This may be related to the personal experience of acute or persistent COVID-19 symptoms (66). These physical symptoms and isolation requirements could have further disrupted various aspects of the individuals’ functioning, like social interaction and study progress. As such, the level of intrusiveness of COVID-19 with stress from physical conditions was stronger and more pervasive in the COVID network.

Stress from FFCs was significantly more connected with appetite problems in the COVID network. Appetite was usually reduced during both acute and post-acute COVID-19 infection (67). However, overeating was a common stress-coping behavior, and interpersonal stress was also a well-known etiological factor for overeating (20, 68). Stress-eating was shown to be more common during quarantine and lockdown (69). It was unknown how these factors interplayed and contributed to our observations and whether the change in appetite referred to its increase, decrease, or a combination of both. At the time of our study, COVID-19–infected individuals were required to undergo compulsory isolation for at least 14 days in designated facilities, meaning that they would have experienced a long period of physical, social, and emotional distancing. Despite the widespread use of virtual communication platforms, interpersonal conflicts could be more difficult to be resolved and, thus, a stronger effect in change in appetite (70).

Our findings demonstrated the exceptional importance of perceived social support and stress from FFCs in deactivating and activating the network of symptoms in COVID network. Unique to the COVID network, stress from FFCs formed a separate community with SSFr and SSFm and two key depressive symptoms: negative cognition and suicidal ideation. Stress from FFCs and negative cognitions ranked among the top in the value of bridge expected influence, indicating its importance in activating other communities of symptoms. Although not statistically significant, SSFr had a stronger negative correlation to suicidal ideation in the COVID network. Earlier studies suggested that perceived social support negatively related to anxiety or depressive symptom levels, assessed as summative scores (14, 71) or as general psychological well-being (37, 59). A few studies looked into the associations of general perceived stress (72), specific stressors, like financial stress (14) or academic stress (73), to mental health. So far, there has been no earlier study looking into how COVID-19 infection status may affect the relationships among perceived social support, interpersonal conflicts, and psychopathological symptoms. However, the link between the lack of social support with hopelessness and poor quality of life was reported in different patient groups (74, 75). As aforementioned, social support that involved fear and anxiety and inability to meet individuals’ need would lose its protective effect (64). For those with COVID-19, they might have accepted the fact of having the infection with mostly mild symptoms and were less affected by the potentially negative influence from social support that involves fear and anxiety. This was consistent with the loss of all positive correlations between social support and stress symptoms in the COVID network. The quality and dimension of social support perceived and the needs of those infected could also be different. Material and information support that targeted the materialistic needs and physical symptoms would have been highly valued by those with COVID-19 infection.

In the overall network, the central symptoms were depressed mood and uncontrollable worries, an observation consistent with previous studies (4, 76–80) during the earlier and later waves of the pandemic. Cheung et al. (2021) showed that guilt or negative cognition was the central symptoms in our local population in, 2020, which might be related to the exclusion of anxiety or stress symptoms, the sampling of general population, and the larger influence from the large-scale protests in, 2019 (81). The bridging symptoms were concentration and psychomotor problems or irritability (strongly correlated with each other), restlessness, and depressed mood. These were comparable to previous studies not limited to university students (4, 6, 76, 82–84) and shared similarities to nervous tension and hyperarousal symptoms as hypothesized in the tripartite model of anxiety and depression (85). Drawing upon previous research in understanding anxiety and depressive symptoms, anxiety symptoms in depression predicts poor treatment outcomes (86). Although comorbid anxiety and depression is highly common and share common risk factors, anxiety symptoms generally surfaced in adolescence and early adulthood preceding the onset of depression (87). Having increased awareness and early identification of these bridging symptoms are important for interventions before a full-blown depressive episode develops. Interventions like mindfulness-based practices have been shown to reduce irritability and improve concentration (88, 89) and were associated with reduced depressive and anxiety symptoms during COVID-19 pandemic (90).

The strengths in our study were the inclusion of the most common psychopathological symptoms measured using standardized instruments, the unique timing of data collection that allows the investigation of specific stressors and psychopathology in this highly stressful period, and the ability to compare these networks in the COVID-19–infected and non-infected groups recruited using a unified method. Several limitations should be noted. Firstly, COVID-19 infection status was based on self-reports only. Although rapid test kits were widely available and the public was highly encouraged to undergo self-testing, the decisions to undergo self-testing were unlikely random and the results could be inaccurate. Individuals reporting an absence of infection could have had it without being tested, had a false negative result, or experienced fewer physical symptoms. The differences observed between the COVID network and no_COVID network could have been overestimated. Secondly, the timing of infection was not recorded. Increased psychiatric symptoms had been reported in both acute and post-infective period and lethargy and sleep problems were common in the post-COVID-19 syndrome (91). As of the end of the sampling period, 92% of all COVID-19 cases reported locally were reported in the preceding 40 days. It would be reasonable to suggest the network approximated the circumstances during the acute and 1-month post-infective period. Given the significant differences in the relationships of the stressors to psychopathological symptoms between the COVID and no_COVID network and the ever-evolving pandemic, the symptom network could be different along the stages of infection and pandemic. Thirdly, stressors and social support were not measured using standardized instruments. Validated dimensional measures of social support like instrumental, emotional, or informational support were not evaluated. Fourthly, the impact of academic stress was not explored. Finally, the non-randomized sampling and cross-sectional nature of the study limit the generalisability of the results and the causality among the factors studied.

In conclusion, network analysis was a useful approach for researchers to understand how psychopathology evolved with multiple external stressors during the COVID-19 pandemic. Our results suggested that, at the peak of the pandemic, vigorously promoting practical methods to prevent, handle, or resolve interpersonal conflicts especially in those infected with COVID-19; facilitating stress-buffering social support in times of social distancing measures; and actively teaching techniques to manage concentration problems, irritability, and restlessness are important interventions that could mitigate mental health distress of college students. In hindsight, most of the mental health promotion strategies during COVID-19 were developed on the basis of general stress management and psychoeducation. Future studies would be necessary to test out whether, indeed, targeted interventions are more useful. Although COVID-19 is no longer a global public health emergency, these preliminary findings should be considered for future pandemic preparedness.

## Data availability statement

The raw data supporting the conclusions of this article will be made available by the authors, without undue reservation.

## Ethics statement

The studies involving humans were approved by Institutional Review Board of the University of Hong Kong/Hospital Authority Hong Kong West Cluster. The studies were conducted in accordance with the local legislation and institutional requirements. The participants provided their written informed consent to participate in this study.

## Author contributions

WChang: Conceptualization, Funding acquisition, Methodology, Supervision, Writing – review & editing. CL: Formal Analysis, Writing – original draft. KNC: Data curation, Investigation, Methodology, Writing – review & editing. SW: Conceptualization, Methodology, Writing – review & editing. HW: Investigation, Methodology, Project administration, Writing – review & editing. HL: Investigation, Methodology, Project administration, Writing – review & editing. YS: Investigation, Methodology, Project administration, Writing – review & editing. SF: Investigation, Methodology, Project administration, Writing – review & editing. SC: Investigation, Methodology, Project administration, Writing – review & editing. KKC: Methodology, Writing – review & editing. PC: Methodology, Writing – review & editing. KL: Methodology, Writing – review & editing. WChan: Methodology, Writing – review & editing.
