# Supplementary material for: Relationships between psychopathological symptoms, pandemic-related stress, perceived social support, and COVID-19 infection history: a network analysis in Chinese college students
Source: Front Psychiatry. 2024 Feb 2;15:1340101. doi: 10.3389/fpsyt.2024.1340101 (PMC10873916; doi:10.3389/fpsyt.2024.1340101)
Supplement: Supplementary file 1 [file Table_1.docx]

**Supplementary Materials**

**Table S1.** Psychiatric symptom score distribution and predictability

**Table S2.** Psychiatric symptom score distribution in those with and without a report of COVID-19 infection.

**Table S3.** Partial correlation matrix of the variables for the whole sample

**Figure S1.** Bootstrapped confidence intervals of estimated edge-weights for the estimated network

**Figure S2.** Bootstrapped difference test between non-zero edge-weights in the estimated network.

**Figure S3.** Bootstrapped difference test on node expected influence of the estimated network.

**Figure S4.** Stability of centrality indexes using case-dropping bootstrap.

**Table S4.** Partial correlation matrix of the variables for the subsample with COVID-19 infection history

**Table S5.** Partial correlation matrix of the variables in for the subsample without COVID-19 infection history

**Table S6.** Fit indexes of confirmatory factor analysis of the communities identified in exploratory group analysis of the overall, COVID and no_COVID network models.

**Supplementary Table S1.** Psychiatric symptom score distribution and predictability (N=1359)

| Items | Mean (SD) | Predictability |
| --- | --- | --- |
| *PHQ-9* |  |  |
| PHQ1 – depressed mood | 1.34 (0.90) | 0.59 |
| PHQ2 – anhedonia | 1.13 (0.87) | 0.66 |
| PHQ3 – sleep problem | 1.23 (1.01) | 0.39 |
| PHQ4 – lethargy | 1.52 (0.95) | 0.57 |
| PHQ5 – appetite problem | 0.83 (0.93) | 0.41 |
| PHQ6 – negative cognition | 0.91 (0.99) | 0.56 |
| PHQ7 – concentration problem | 1.12 (0.99) | 0.53 |
| PHQ8 – psychomotor problem | 0.68 (0.88) | 0.49 |
| PHQ9 – suicidal idea | 0.31 (0.67) | 0.36 |
| Total score | 9.07 (6.00) | 0.51 |
| *GAD-7* |  |  |
| GAD1 – anxious mood | 1.26 (0.90) | 0.66 |
| GAD2 – uncontrollable worries | 1.02 (0.98) | 0.75 |
| GAD3 – generalized worries | 1.13 (0.99) | 0.72 |
| GAD4 – difficulty relaxing | 1.19 (1.00) | 0.66 |
| GAD5 – restlessness | 0.89 (0.95) | 0.67 |
| GAD6 – irritability | 0.89 (0.96) | 0.55 |
| GAD7 – anticipatory anxiety | 0.71 (0.89) | 0.56 |
| Total score | 7.30 (5.62) | 0.65 |
| *IES-6* |  |  |
| IES1 – frequent reminders of COVID | 1.28 (1.06) | 0.57 |
| IES2 – uncontrollable thoughts about COVID | 1.25 (1.15) | 0.63 |
| IES3 – avoid thinking about COVID | 1.03 (1.05) | 0.44 |
| IES4 – lots of feelings not yet dealt with | 1.17 (1.11) | 0.54 |
| IES5 – concentration problem | 1.75 (1.23) | 0.61 |
| IES6 – on-guard | 1.55 (1.19) | 0.42 |
| Total score | 8.03 (5.29) | 0.54 |
| COVID, coronavirus; GAD, Generalized Anxiety Disorder Scale; IES, Impact of Event Scale; PHD, Patient Health Questionnaire; SD, standard deviation. | | |

**Supplementary Table S2.** Psychiatric symptom score distribution in those with and without a report of COVID-19 infection.

|  | Mean (SD) | |
| --- | --- | --- |
| Items | COVID (n=319) | No_COVID (n=1040) |
| *PHQ-9* |  |  |
| PHQ1 – depressed mood | 1.48 (0.90) | 1.30 (0.90) |
| PHQ2 – anhedonia | 1.21 (0.87) | 1.10 (0.87) |
| PHQ3 – sleep problem | 1.34 (0.97) | 1.20 (1.02) |
| PHQ4 – lethargy | 1.66 (0.89) | 1.48 (0.96) |
| PHQ5 – appetite problem | 0.91 (0.94) | 0.80 (0.93) |
| PHQ6 – negative cognition | 1.03 (1.04) | 0.87 (0.96) |
| PHQ7 – concentration problem | 1.23 (0.99) | 1.09 (0.98) |
| PHQ8 – psychomotor problem | 0.77 (0.90) | 0.65 (0.87) |
| PHQ9 – suicidal idea | 0.37 (0.70) | 0.29 (0.66) |
| Total score | 9.99 (5.72) | 8.79 (6.06)* |
| *GAD-7* |  |  |
| GAD1 – anxious mood | 1.39 (0.89) | 1.22 (0.90) |
| GAD2 – uncontrollable worries | 1.14 (1.04) | 0.98 (0.96) |
| GAD3 – generalized worries | 1.26 (1.04) | 1.08 (0.97) |
| GAD4 – difficulty relaxing | 1.28 (1.00) | 1.16 (1.00) |
| GAD5 – restlessness | 0.96 (0.98) | 0.87 (0.94) |
| GAD6 – irritability | 1.23 (0.91) | 1.06 (0.95) |
| GAD7 – anticipatory anxiety | 0.82 (0.95) | 0.68 (0.86) |
| Total score | 8.08 (5.86) | 7.05 (5.52)* |
| *IES-6* |  |  |
| IES1 – frequent reminders of COVID | 1.32 (1.05) | 1.27 (1.06) |
| IES2 – uncontrollable thoughts about COVID | 1.30 (1.12) | 1.24 (1.16) |
| IES3 – avoid thinking about COVID | 1.03 (1.10) | 1.03 (1.17) |
| IES4 – lots of feelings not yet dealt with | 1.33 (1.14) | 1.12 (1.09) |
| IES5 – concentration problem | 1.92 (1.21) | 1.70 (1.24) |
| IES6 – on-guard | 1.57 (1.19) | 1.58 (1.19) |
| Total score | 8.46 (5.11) | 7.89 (5.34) |

COVID, coronavirus; GAD, Generalized Anxiety Disorder Scale; IES, Impact of Event Scale; PHD, Patient Health Questionnaire; SD, standard deviation.

**p*<0.01

**Supplementary Table S3.** Partial correlation matrix of the variables for the whole sample.


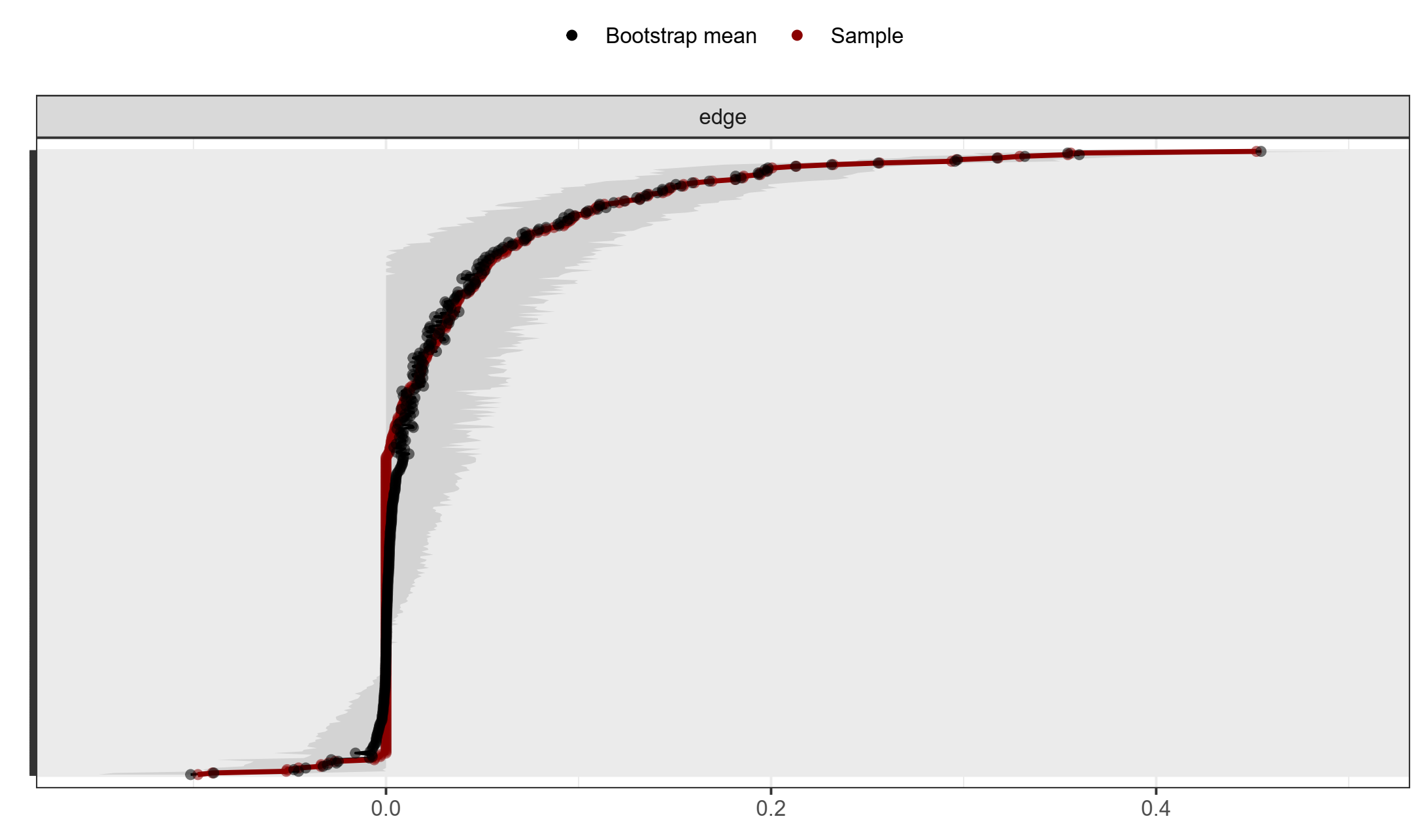


**Supplementary Figure S1.** Bootstrapped confidence intervals of estimated edge-weights for the estimated network. The red line denotes the sample values and the grey area denotes the bootstrapped CIs. Edges are ordered from the highest to the lowest edge-weight


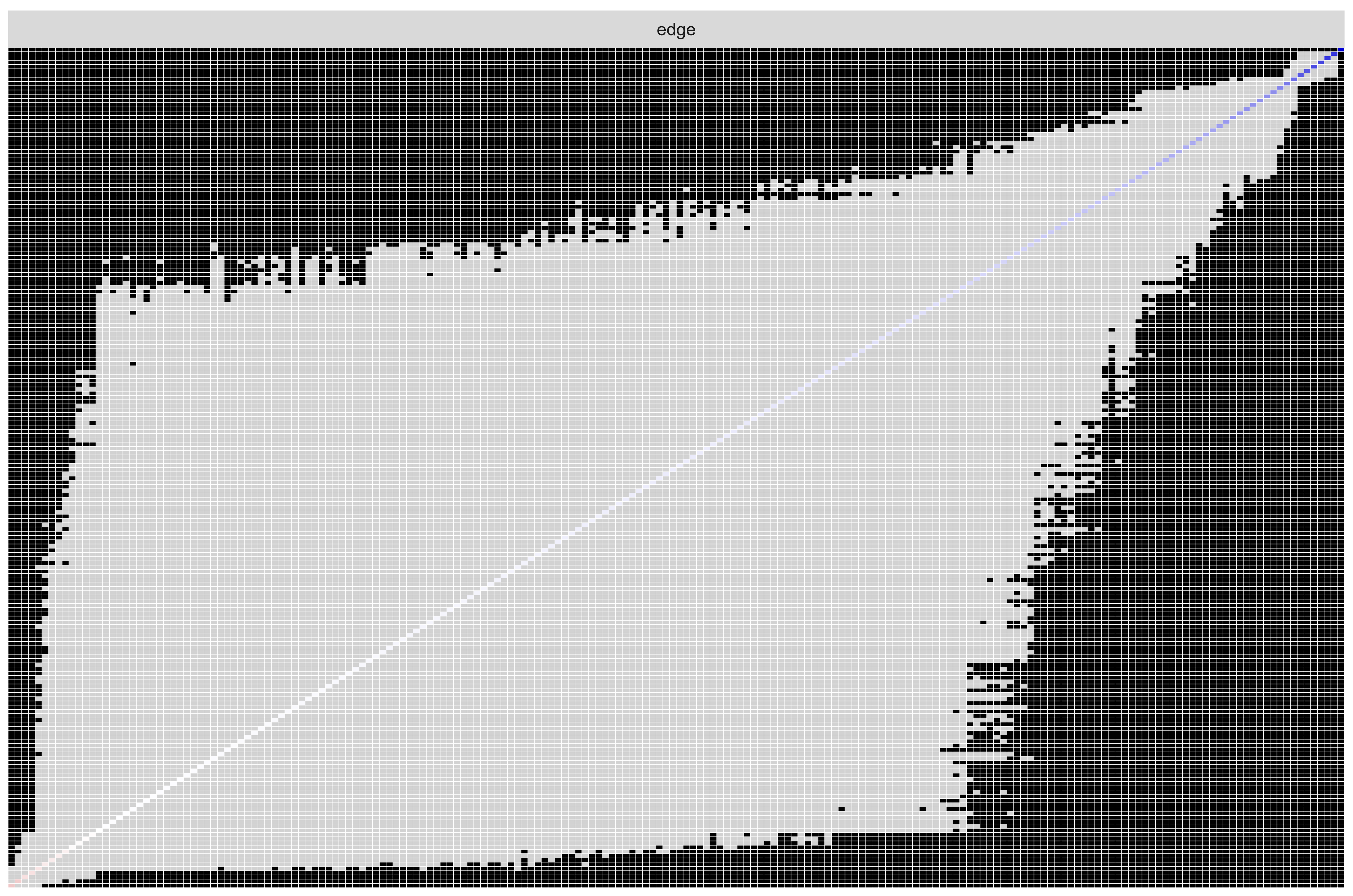


**Supplementary Figure S2.** Bootstrapped difference test between non-zero edge-weights in the estimated network. Gray boxes indicate edges that do not differ significantly from one-another, while black boxes indicate significant differences between edges.


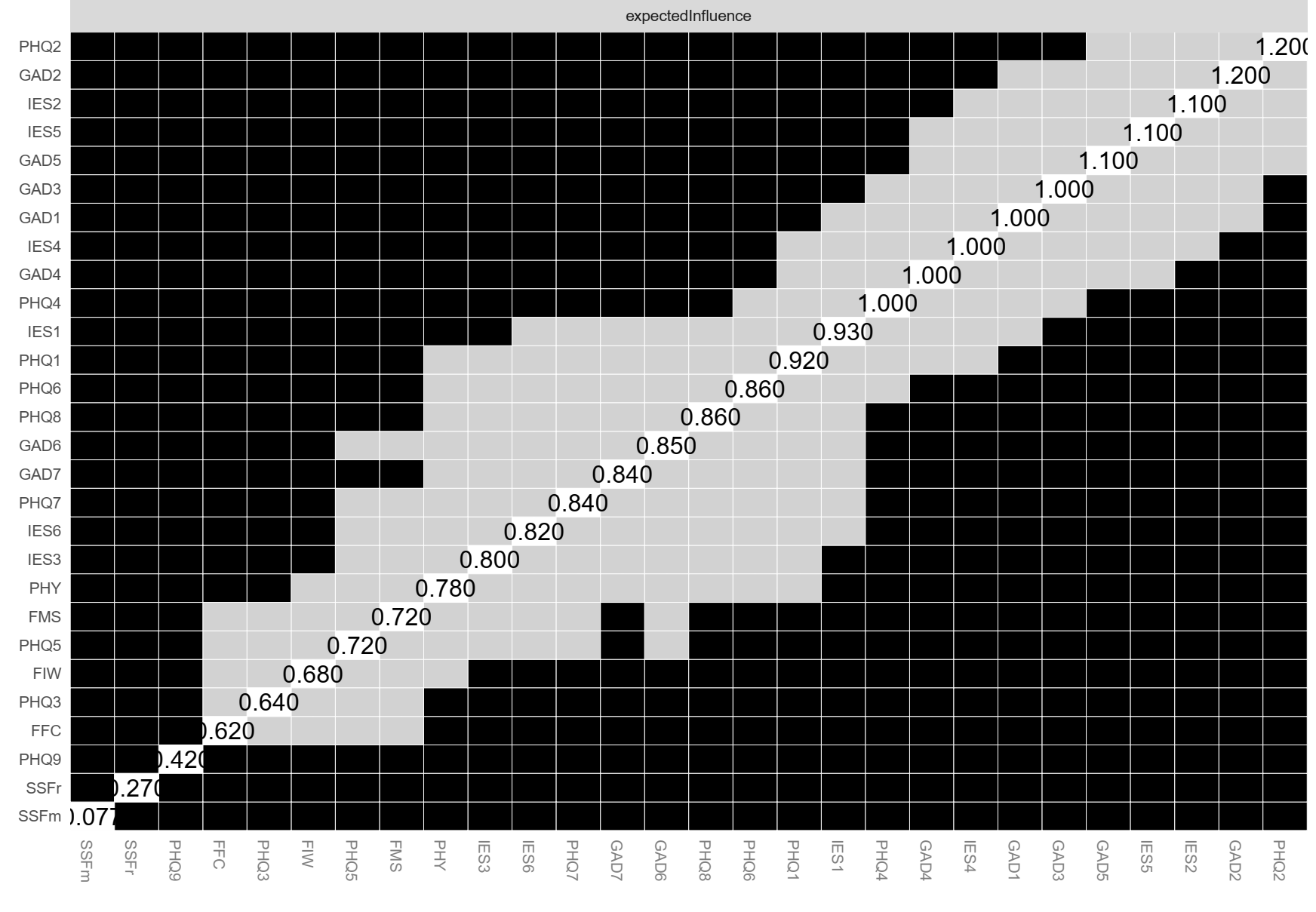


**Supplementary Figure S3.** Bootstrapped difference test on node expected influence of the estimated network. Gray boxes indicate nodes that do not differ significantly from one-another, while black boxes indicate significant differences between nodes. White boxes in the centrality plot show the value of node expected influence.


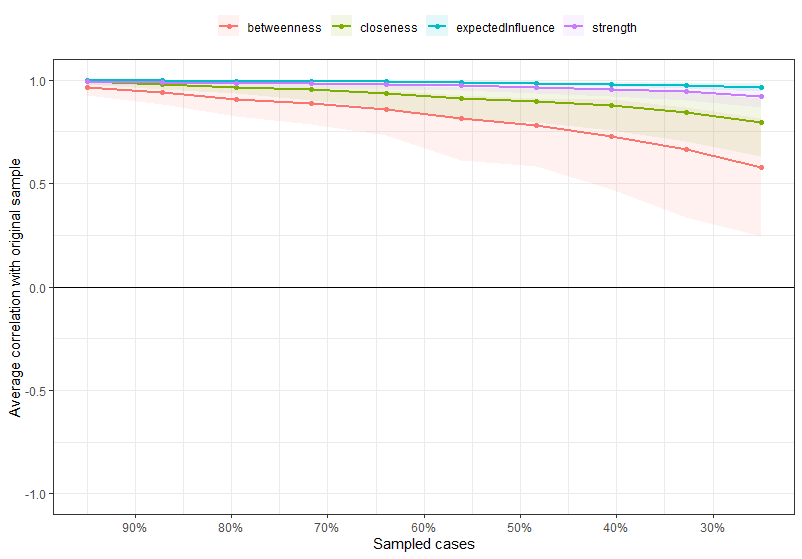


**Supplementary Figure S4.** Stability of centrality indexes using case-dropping bootstrap. The x-axis represents the percentage of cases from the original sample included in each step. The y-axis represents the average correlations between the centrality indices from the original network and those re-estimated from the specified proportion of cases sampled.

**Supplementary Table S4.** Partial correlation matrix of the variables for the subsample with COVID-19 infection history (COVID network)

**Supplementary Table S5.** Partial correlation matrix of the variables for the subsample without COVID-19 infection history (no_COVID network)

**Supplementary Table S6.** Fit indexes of confirmatory factor analysis of the communities^a^ identified in exploratory group analysis of the overall, COVID and no_COVID network models.

| Fit indexes^b^ | Overall network | COVID network | No COVID network |
| --- | --- | --- | --- |
| Chi-square/degree of freedom (χ2/df) | 4.489 | 2.162 | 4.129 |
| RMSEA | 0.051 | 0.061 | 0.055 |
| SRMR | 0.045 | 0.066 | 0.049 |
| CFI | 0.994 | 0.992 | 0.993 |

Abbreviation: CFI = comparative fit index; RMSEA = root mean square error of approximation; SRMR = standardized root mean square residual

^a^ Communities detected for each network:

Overall network: [1] "PHQ1" "PHQ2" "PHQ3" "PHQ4" "PHQ5" "PHQ6" "PHQ9" [2] "GAD1" "GAD2" "GAD3" "GAD4" "GAD5" "GAD6" "GAD7" [3] "IES1" "IES2" "IES3" "IES4" "IES6" [4] "FIW" "PHY" "FMS" "FFC" [5] "PHQ7" "PHQ8" "IES5" [6] "SSFm" "SSFr"

COVID network: [1] "PHQ1" "PHQ2" "PHQ3" "PHQ4" "PHQ5" "PHQ7" "PHQ8" "GAD6" "IES5" [2] "IES1" "IES2" "IES3" "IES4" "IES6" "FIW" "PHU" "FMS" [3] "GAD1" "GAD2" "GAD3" "GAD4" "GAD5" "GAD7" [4] "PHQ6" "PHQ9" "SSFm" "SSFr" "FFC"

No_COVID network: [1] "PHQ1" "PHQ2" "PHQ3" "PHQ4" "PHQ5" "PHQ6" "PHQ7" "PHQ8" "PHQ9" "IES5" [2] "GAD1" "GAD2" "GAD3" "GAD4" "GAD5" "GAD6" "GAD7" [3] "IES1" "IES2" "IES3" "IES4" "IES6" [4] "FIW" "PHY" "FMS" "FFC" [5] "SSFm" "SSFr"

^b^ Interpretation of fitness indexes: Chi-square to degree of freedom ratio ranged 2-5 is considered acceptable; RMSEA ≤ 0.05 indicates good fit, ≤ 0.08 indicates acceptable fit; SRMR ≤ 0.05 indicates good fit, ≤ 0.08 indicates acceptable fit; CFI ≥ 0.90 indicates good fit.

Reference: Hooper D, Coughlan J & Mullen MR. Structural Equation Modelling: Guidelines for Determining Model Fit. *Electron. J. Bus. Res. Methods* 2008; 6: 53-60.
